# Performance of the ABC-bleeding risk score for assessing major bleeding risk in Chinese patients with atrial fibrillation on oral anticoagulation therapy: A real-world study

**DOI:** 10.3389/fcvm.2022.1019986

**Published:** 2022-11-03

**Authors:** Yu-Feng Wang, Chao Jiang, Liu He, Cun-Ying Pu, Xin Du, Cai-Hua Sang, De-Yong Long, Ri-Bo Tang, Jian-Zeng Dong, Chang-Sheng Ma

**Affiliations:** ^1^Department of Cardiology, National Clinical Research Centre for Cardiovascular Diseases, Beijing Anzhen Hospital, Capital Medical University, Beijing, China; ^2^Roche Diagnostics (Shanghai) Limited, Medical and Scientific Affairs, Shanghai, China; ^3^Heart Health Research Center, Beijing, China; ^4^Faculty of Medicine, The George Institute for Global Health, University of New South Wales, Sydney, NSW, Australia; ^5^Centre for Cardiovascular Diseases, The First Affiliated Hospital of Zhengzhou University, Zhengzhou University, Zhengzhou, Henan, China

**Keywords:** atrial fibrillation, ABC-bleeding risk score, HAS-BLED score, major bleeding, anticoagulation, real-world study, GDF-15

## Abstract

**Objective:**

To evaluate performance of the ABC (Age, Biomarkers, Clinical history)-bleeding risk score in estimating major bleeding risk in Chinese patients with atrial fibrillation (AF) on oral anticoagulation (OAC) therapy in real-world practice.

**Methods:**

Data were collected from the Chinese Atrial Fibrillation Registry study (CAFR). Patients were stratified into low-, medium-, and high-risk groups based on ABC-bleeding risk score with 1-year major bleeding risk (<1%, 1–2%, and > 2%) and modified HAS-BLED score (≤1, 2, and > 2 points). Cox proportional-hazards (Cox-PH) models were used to determine the association of major bleeding incidence with bleeding scores. Harrell’s C-index of the two scores were compared. Net reclassification improvement (NRI) and integrated discrimination improvement (IDI) at 1 year were employed to evaluate the reclassification capacity. The calibration curve was plotted to compare the predicted major bleeding risk using ABC-bleeding risk score with the observed annualized event rate. The decision analysis curves (DCA) were performed to show the clinical utilization of two scores in identifying major bleeding events.

**Results:**

The study included 2,892 AF patients on OAC therapy. After the follow-up of 3.0 years, 48 patients had major bleeding events; the incidence of a bleeding event in the low-, medium-, and high-risk groups according to ABC-bleeding risk score was 0.31% (reference group, HR = 1.00),0.51% (HR = 1.83, 95%CI: 0.91–3.69, *P* = 0.09), and 1.49% (HR = 4.92, 95%CI: 2.34–10.30, *P* < 0.001), respectively. Major bleeding incidence had an independent association with growth differentiation factor 15 (GDF-15) level (HR = 2.16, 95%CI: 1.27–3.68, *P* = 0.005) after adjusting components of the HAS-BLED score and cTnT-hs level. The ABC-bleeding score showed a Harrell’s C-index of 0.67 (95%CI: 0.60–0.75) in estimating major bleeding risk, which was non-significant compared to the modified HAS-BLED score (0.67 vs. 0.63; *P* = 0.38). NRI and IDI also revealed comparable reclassification capacity of ABC-bleeding risk score compared with HAS-BLED score (14.6%, 95%CI: −10.2%, 39.4%, *P* = 0.25; 0.2%, 95%CI −0.1 to 0.9%, *P* = 0.64). Cross-tabulation of the two scores showed that the ABC-bleeding score outperformed the HAS-BLED score in identifying patients with a high risk of major bleeding. The calibration curve showed that the ABC-bleeding risk score overestimated the observed major bleeding risk. DCA did not show any difference in net benefit when using either of the scores.

**Conclusion:**

This study verified the value of the ABC-bleeding risk score in assessing major bleeding risk in Chinese patients with AF on OAC therapy in real-world practice. Despite the overestimation of major bleeding risk, ABC-bleeding score performed better in stratifying patients with a high risk than the modified HAS-BLED score. Combining the two scores could be a clinically practical strategy for precisely stratifying AF patients, especially those at a high risk of major bleeding, and further supporting the optimization of OAC treatment.

## Introduction

Atrial fibrillation (AF) is the most common sustained arrhythmia in China with a weighted prevalence of 1.8% [95% confidence interval (CI): 1.7–1.9] (1). Patients with AF have an approximately fivefold increased risk of ischemic stroke, and international guidelines recommend oral anticoagulation (OAC) therapy for stroke prevention (2, 3). However, anticoagulation might result in major bleeding events, which sometimes are even fatal. Therefore, the development of bleeding risk assessment tools is of great importance for decisions making in anticoagulation treatment.

The HAS-BLED [Hypertension, Abnormal renal/liver function, Stroke, Bleeding history or predisposition, Labile international normalized ratio, Elderly (>65 years), Drugs/alcohol concomitantly] score was developed and validated to assess major bleeding risk in patients with AF on OAC therapy (4–6). The 2020 European Society of Cardiology (ESC) AF guidelines recommended the HAS-BLED score to assess major bleeding risk (3). However, as based solely on clinical factors, the HAS-BLED score has only a modest predictive ability for high-risk patients (5).

Recently, several biomarkers have shown the potential to reflect cardiovascular and renal physiology, coagulation, and inflammatory activity, which can determine AF prognosis (7–9).

Growth differentiation factor 15 (GDF-15), secreted by a broad range of cells upon hypoxia and oxidative stress, is a marker of cellular aging and inflammatory activity, as well as a major risk indicator of hemorrhages in patients with AF treated with OAC, even adjusted by the clinical components of HAS-BLED score and other biomarkers (10, 11). In 2016, Hijazi et al. reported a novel biomarker-based tool, the ABC (Age, Biomarkers, Clinical History)-bleeding risk score. The components of the ABC-bleeding risk score include age, GDF-15, high-sensitive cardiac troponin T (cTnT-hs), hemoglobin, and history of bleeding (12). The ABC-bleeding risk score was externally validated and calibrated through several large AF clinical trials (13), and thus is considered more favorable in evaluating major bleeding risk than the HAS-BLED risk score owing to its higher Harrell’s C-index suggesting better predictive performance (12, 14). However, a real-world investigation raised a contrary conclusion that the HAS-BLED score outperformed the ABC-bleeding score in estimating major bleeding events (15). Therefore, it is still debatable whether the ABC-bleeding score is better than the HAS-BLED score for predicting major bleeding, and more evidence from real-world practice is required for further confirmation.

Considering the large population of patients with AF on OAC therapy in China, we aimed to validate the predictive value of the ABC-bleeding risk score and assess how it compared to the HAS-BLED score in Chinese patients with AF receiving OAC based on real-world evidence.

## Materials and methods

Our study enrolled patients between 2014 and 2018 from the Chinese Atrial Fibrillation Registry study (CAFR), a prospective, multicenter, hospital-based, ongoing registry study that includes Chinese patients with AF (16). CAFR consecutively enrolled patients with AF from 31 tertiary and non-tertiary hospitals in Beijing, China, with regular follow-ups every 6 months. Data related to AF, including demographics, medical history, symptoms and signs, comorbidities, medical treatment, physical examination, and biochemical tests, were collected. A specialized follow-up team independently recorded clinical events such as major bleeding, stroke, myocardial infarction, and other cardiovascular events.

This study was approved by the Human Research Ethics Committee of Beijing Anzhen Hospital and conducted in accordance with the Declaration of Helsinki.

### Study population

A total of 2,892 patients were included in the study and observed with a mean follow-up of 3 years. Patients aged ≥ 18 years who had received OAC treatment lasting at least 3 months were enrolled. Patients were excluded if they met any of the following criteria: valvular AF, including any mechanical valves, or moderate to severe mitral stenosis; unstable conditions: onset of acute coronary syndrome, acute heart failure, stroke, transient ischemic attacks (TIA), and major bleeding events within the 6 months before baseline; data missing during follow-up.

### Data collection

Baseline data were collected, including demographic characteristics, comorbidities, OAC treatment received, and biomarker measurements. Major bleeding risk was evaluated by the ABC-bleeding risk and the modified HAS-BLED scores that international normalized ratio (INR) lability was not included as time in therapeutic range (TTR) data were unavailable in this study. Based on the ABC-bleeding risk score predicting 1-year major bleeding risk, patients were stratified into three groups: low-risk (<1%), medium-risk (1–2%), and high-risk (>2%). The modified HAS-BLED score was also calculated to stratify patients into three risk groups: low (0–1 point), medium (2 points), and high (>2 points).

### Biochemical samples and laboratory analysis

Three biomarkers were measured in the ABC-bleeding risk score (GDF-15, cTnT-hs, and hemoglobin). EDTA-anticoagulated blood samples were collected from the enrolled patients. Plasma samples were obtained following centrifugation and stored at −70°C until being analyzed centrally. GDF-15 level was analyzed using the Elecsys^®^ GDF-15 assay (Roche Diagnostics International Ltd., Rotkreuz, Switzerland) with the same standardization as other routine reagents on cobas^®^ e 801 analytical unit (Roche Diagnostics International Ltd., Rotkreuz, Switzerland). Other biomarkers included in the ABC-bleeding risk score were measured using the previously published method (10, 11). All analyses were conducted according to manufacturer instructions.

### Clinical outcomes

The primary endpoint of this study was major bleeding events defined according to the 2005 International Society on Thrombosis and Hemostasis criteria: fatal bleeding or symptomatic bleeding in a critical anatomical site (intracranial, intraspinal, intraocular, retroperitoneal, intraarticular, pericardial, or intramuscular with compartment syndrome), and/or bleeding causing a fall in hemoglobin ≥ 20 g/L, or transfusion of ≥ 2 units of whole blood or red blood cells (17).

### Statistical analysis

Baseline characteristics were described and stratified into the three ABC-bleeding risk score levels. Continuous variables were described as mean ± standard deviation (SD) or median with interquartile range (IQR), depending on the data distribution.

Cox-proportional hazard (Cox-PH) regression models were conducted to assess the association between the risk levels identified by the ABC-bleeding risk and the modified HAS-BLED scores and major bleeding incidence. Kaplan-Meier (K-M) curves showing the probability of major bleeding in each ABC-bleeding 1-year and modified HAS-BLED risk level were plotted. Log-rank test was conducted to compare the survival distributions between groups. Harrell’s C-index was calculated to evaluate the discriminatory performance of the ABC-bleeding risk and the modified HAS-BLED scores. Net reclassification improvement (NRI), and integrated discrimination improvement (IDI) at 1 year, the positive value of which can indicate an improvement in risk prediction (18), were used to assess the reclassification performance of the ABC-bleeding risk score in predicting 1-year major bleeding risk compared with the modified HAS-BLED score. The calibration curve of ABC-bleeding risk score was plotted by comparing the predicted one-year risk with observed annualized event rate. The decision curves analysis (DCA) was employed to evaluate the net benefit of using one score to identify major bleeding events. Sensitivity analysis was further conducted according to the anticoagulation types and by the exclusion of those with antiplatelet therapy.

In this study, a one-side *P*-value of < 0.025 was considered statistically significant for the Cox-PH regression model, and *P* < 0.05 was considered statistically significant for all other analyses. All analyses were conducted using R 4.0.0 and SAS 9.4 statistical software.

## Results

### Baseline characteristics

Demographic and baseline characteristics of the three patient groups stratified by ABC-bleeding risk score are summarized in Table 1. In total, 2,892 patients were included in the cohort, with a mean age of 59.9 (SD: 10.70) years and 68.0% male. The mean age of patients decreased from 72.72 (SD: 8.21) years in the high-risk group to 53.18 (SD: 8.66) years in the low-risk group. Paroxysmal AF was the most common type of AF, present in over 60% of patients in each group. Compared with patients in the medium- and low-risk groups, high-risk patients had a lower estimated glomerular filtration rate and were more likely to suffer from multiple cardiovascular comorbidities, particularly hypertension, coronary artery disease, and heart failure. Non-vitamin K antagonist oral anticoagulants (NOACs) were the most common anticoagulation therapies that were used to treat 50–70% of patients across the three groups. Median GDF-15 level increased from 783.00 ng/L (IQR: 619.00, 983.50) in the low-risk group to 2,075.00 ng/L (IQR: 1532.00, 2773.00) in the high-risk group. The CHA_2_DS_2_-VASc (a clinical prediction rule used for estimating the risk of stroke in patients with AF) and the modified HAS-BLED scores in each group showed the same trend of increasing with a higher risk level based on the ABC-bleeding risk score.

**TABLE 1:** Demographic and baseline characteristics stratified by the ABC-bleeding risk score.

	Risk level		
	
	High (*N* = 301)	Medium (*N* = 1,084)	Low (*N* = 1,507)
**Demographics**			
Mean age, years, (SD)	72.72 (8.21)	65.79 (6.29)	53.18 (8.66)
Male, *n* (%)	166 (55.1)	664 (61.3)	1,136 (75.4)
Mean BMI, kg/m^2^, (SD)	25.23 (3.70)	25.46 (3.46)	26.06 (3.57)
AF type, *n* (%)			
New onset	1 (0.3)	4 (0.4)	2 (0.1)
Paroxysmal	197 (65.4)	713 (65.8)	970 (64.4)
Persistent/permanent	103 (34.2)	367 (33.9)	535 (35.5)
Mean eGFR (CKD-EPI), mL/min/1.73 m^2^, (SD)	73.76 (18.59)	83.74 (13.92)	94.54 (13.58)
Current smoking, *n* (%)	24 (8.0)	97 (8.9)	205 (13.6)
Current alcohol consumption, *n* (%)	20 (6.6)	101 (9.3)	211 (14.0)
**Comorbidities, *n* (%)**			
Coronary artery disease	61 (20.3)	175 (16.1)	119 (7.9)
Peripheral arterial disease	6 (2.0)	7 (0.6)	4 (0.3)
Hypertension	221 (73.4)	726 (67.0)	715 (47.4)
Heart failure	31 (10.3)	62 (5.7)	52 (3.5)
Ischemic stroke or TIA	53 (17.6)	154 (14.2)	88 (5.8)
Diabetes mellitus	79 (26.2)	291 (26.8)	215 (14.3)
Previous bleeding	33 (11.0)	65 (5.8)	49 (3.3)
**Medication, *n* (%)**			
OAC therapy			
Warfarin	136 (45.2)	371 (34.2)	443 (29.4)
NOAC	165 (54.8)	713 (65.8)	1,064 (70.6)
Antiplatelet therapy	47 (15.6%)	171 (15.8%)	160 (10.6%)
**Biomarker levels**			
GDF-15, ng/L, medium (IQR)	2075.00 [1532.00, 2773.00]	1281.50 [1061.75, 1602.75]	783.00 [619.00, 983.50]
cTnT-hs, ng/L, median (IQR)	15.60 [11.80, 24.20]	9.54 [7.85, 12.10]	6.56 [5.27, 8.11]
Hemoglobin, g/L, median, (IQR)	136.83 (16.38)	144.75 (13.83)	152.05 (13.72)
CHA_2_DS_2_-VASc, mean ± SD	3.39 (1.47)	2.44 (1.40)	1.12 (1.05)
HAS-BLED, mean ± SD	2.04 (0.91)	1.57 (0.90)	0.92 (0.80)

AF, atrial fibrillation; BMI, body mass index; cTnT-hs, high-sensitive cardiac troponin T; eGFR (CKD-EPI), estimated glomerular filtration rate estimated by the chronic kidney disease epidemiology collaboration equation; GDF-15, growth differentiation factor 15; IQR, interquartile range; NOAC, non-vitamin K antagonist oral anticoagulants, which includes three drugs: rivaroxaban, apixaban, and dabigatran; OAC, oral anticoagulation; SD, standard deviation; TIA, transient ischemic attack.

### Association of ABC-bleeding risk score with major bleeding incidence

The follow-up lasted at least 3 months with a median of 3.0 years. The medium treatment period for patients on warfarin was 293 (IQR: 93, 815) days and 265 (IQR: 98, 447) days for those on NOACs.

In total, 48 major bleeding events occurred during the follow-up. The incidence rate of major bleeding events was 0.51 per 100 person-year. Cox-PH models showed a statistically significant association between major bleeding events and log_2_(GDF-15) [hazard ratio (HR) 2.72, 95% CI: 1.68–4.41, *P* < 0.001; Supplementary Table 1], even after adjusted using the components of HAS-BLED score and level of cTnT-hs (HR 2.16, 95% CI: 1.27-3.68, *P* = 0.005, Supplementary Table 1).

### Risk stratification by ABC-bleeding risk score and how it compares with the modified HAS-BLED score

**FIGURE 1:**
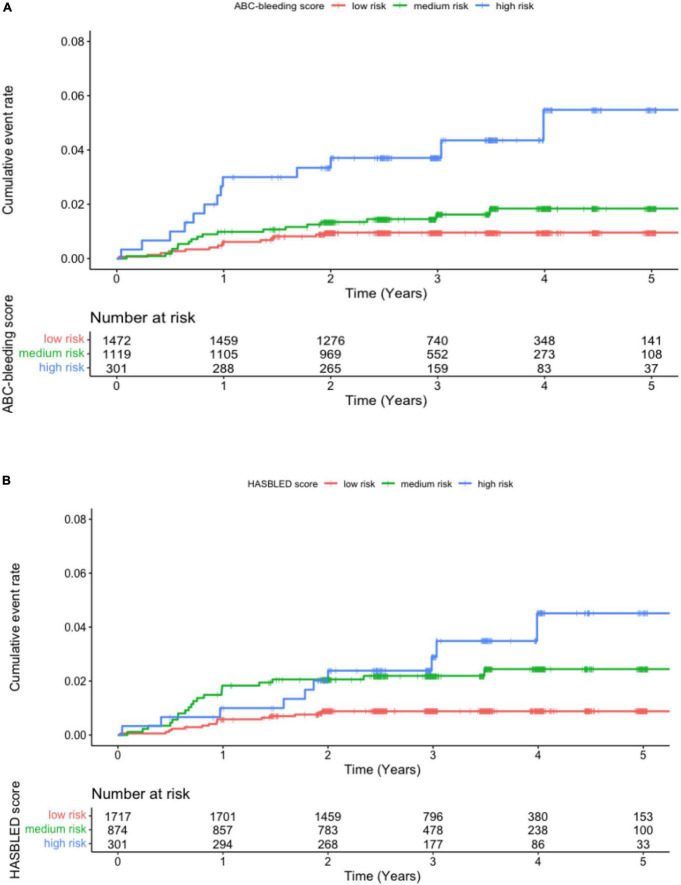
Kaplan-Meier curve for major bleeding in patients at low, medium, and high risk stratified by **(A)** the ABC-bleeding risk and **(B)** the modified HAS-BLED scores.

The 48 major bleeding events were classified into subgroups based on risk levels identified by the ABC-bleeding risk and the modified HAS-BLED scores. The incidence of major bleeding in each group was summarized in Table 2. Specifically, 1,507 (52.11%), 1,084 (37.48%), and 301 (10.41%) patients were identified as low-, medium-, and high-risk, respectively, by the ABC-bleeding risk score, with an increasing incidence of major bleeding from 0.31 (15/48), 0.51 (18/48) to 1.49 (15/48) per 100 person-years, respectively. The Cox-PH regression model showed that the high-risk group identified by the ABC-bleeding risk score had a statistically significant higher major bleeding risk than the low-risk group (HR 4.92, 95% CI: 2.34–10.30, *P* < 0.001). In contrast, the association of risk level with the major bleeding incidence in the medium-risk group failed to reach statistical significance (HR 1.83, 95% CI 0.91–3.69, *P* = 0.09). For patient groups stratified by the modified HAS-BLED score, the Cox-PH regression model revealed that the actual major bleeding risk level was statistically significant in the medium-risk (HR 2.58, 95% CI: 1.32–5.05, *P* = 0.005) and the high-risk groups (HR 3.70, 95% CI 1.67–8.30, *P* = 0.001). Kaplan-Meier curves showed a significant difference in the cumulative incidence rate of major bleeding among the three risk levels stratified by ABC-bleeding and the modified HAS-BLED scores (*P* < 0.001 and *P* = 0.001, respectively, Figure 1). However, cumulative incidence rate curves and log-rank test between each two risk level groups by the modified HAS-BLED score showed that the survival distribution between the median- and high-risk groups did not reach a significant difference (*P* = 0.35, Supplementary Figure 1).

**TABLE 2:** Major bleeding incidence and Cox proportional-hazards regression analysis of risk levels stratified by the ABC-bleeding and the modified HAS-BLED scores.

	ABC-bleeding score risk level			Modified HAS-BLED score risk level		
		
	Low	Medium	High	Low	Medium	High
N (%)	1,507	1,084	301	1,717	874	301
No. of events	15	18	15	15	23	10
Incidence (per 100 person-years)	0.31	0.51	1.49	0.27	0.78	0.97
HR (95% CI)*	−	1.83 (0.91–3.69)	4.92 (2.34–10.30)	–	2.58 (1.32–5.05)	3.70 (1.67–8.30)
*P*-value	−	0.09	<0.001	−	0.005	0.001

CI, confidence interval; HR, hazard ratio.

*HR vs. the low-risk group.

To further evaluate the discriminatory ability of the ABC-bleeding risk score to identify high-risk patients, the low- and medium-risk groups determined by the ABC-bleeding risk score were combined and analyzed using a Cox-PH regression model. Using the combined low + medium risk level as the reference, the high-risk level identified by the ABC-bleeding risk score showed a significantly higher major bleeding risk (HR 3.68, 95% CI: 1.96–6.90, *P* < 0.001). A similar but lower risk was also observed between the high-risk level and low + medium risk level stratified by the modified HAS-BLED score (HR 2.42, 95% CI: 1.20–4.89, *P* = 0.01) (Supplementary Table 2). Corresponding Kaplan-Meier curves also suggested a better performance of the ABC-bleeding risk score than the modified HAS-BLED score in differentiating high-risk patients (Supplementary Figure 2).

### Performance of ABC-bleeding risk score in major bleeding risk and how it compares with the modified HAS-BLED score

As summarized in Table 3, the Harrell’s C-index of the ABC-bleeding risk score was non-significantly higher than that of the modified HAS-BLED score [0.67 (95% CI: 0.60–0.75) vs. 0.63 (95% CI: 0.56–0.70), *P* = 0.38]. The NRI and IDI value at 1-year follow-up for the ABC-bleeding risk score revealed comparable reclassification capacity compared with the modified HAS-BLED score (14.6%, 95%CI: −10.2 to 39.4%, *P* = 0.25; 0.2% 95%CI −0.1 to 0.9%, *P* = 0.64).

**TABLE 3:** Discrimination and reclassification analysis of the ABC-bleeding risk and the modified HAS-BLED scores.

	C-index	95% CI	*P*-value*	NRI at 1 year	*P*- value	IDI at 1 year	*P*-value
ABC-bleeding risk score	0.67	0.60–0.75	0.38	14.6% (−10.2%, 39.4%)	0.25	0.2% (−0.1 to 0.9%)	0.64
Modified HAS-BLED score	0.63	0.56–0.70					

CI, confidence interval; IDI, integrated discrimination improvements; NRI, net reclassification improvement.

*P-value for comparison of ABC-bleeding score with the modified HAS-BLED score.

**FIGURE 2:**
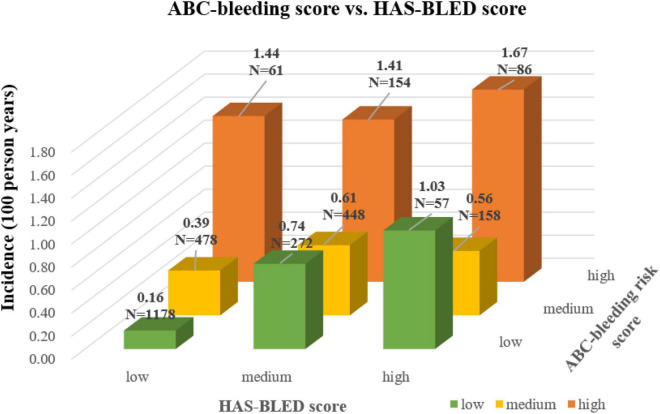
Cross-tabulation showing major bleeding incidence stratified by the ABC-bleeding risk and the modified HAS-BLED scores. The risk level shown by the color of column was assigned based on the modified HAS-BLED score.

To find the underlying relationship between the two scores, the major bleeding incidence in nine patient subgroups cross-tabulated by ABC-bleeding 1-year and the modified HAS-BLED risk levels was shown in Figure 2. ABC-bleeding risk score could further discriminate low-risk patients defined by the modified HAS-BLED score into low-, medium-, and high-risk subgroups with corresponding major bleeding incidences of 0.16, 0.39, and 1.44 per 100 person-years, respectively. Meanwhile, the HAS-BLED score could also stratify low-risk patients determined by the ABC-bleeding score into three different risk levels, with an incidence rate of 0.16, 0.74, and 1.03 per 100 person-years, respectively. Notably, the incidence rate of the three high-risk subgroups stratified by ABC-bleeding score was all higher than 1.4 per 100 person-years, which implied better discrimination of high-risk patients by ABC-bleeding score.

**FIGURE 3:**
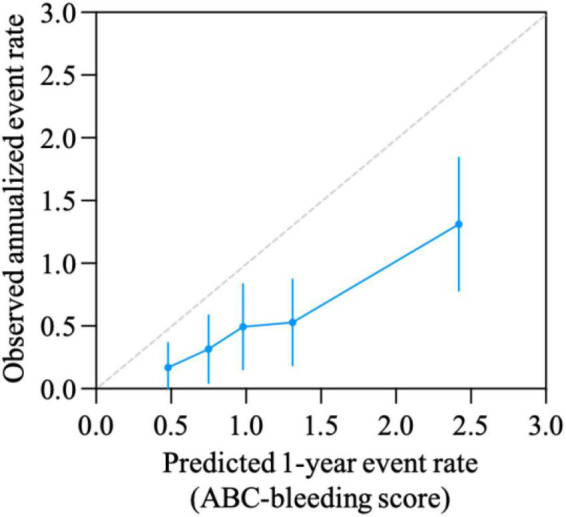
Calibration curve for ABC-bleeding risk score. Calibration was evaluated by comparison of the ABC-bleeding risk score-predicted event rate and the observed annualized events rate.

**FIGURE 4:**
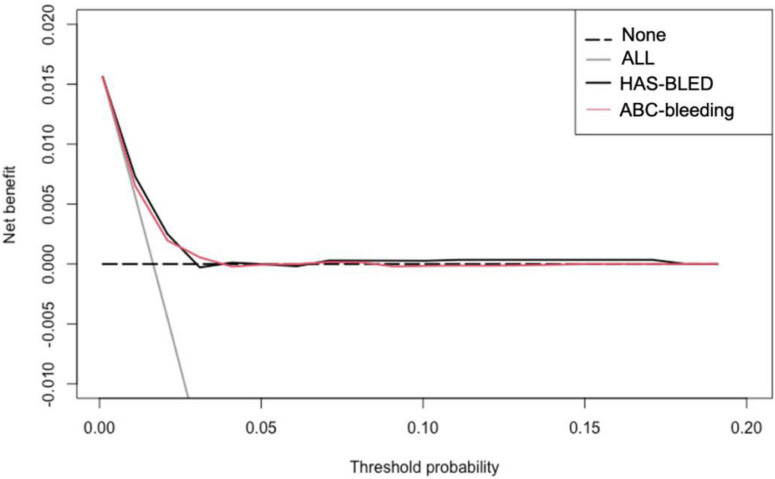
Decision curves for ABC-bleeding risk and HAS-BLED scores. This analysis shows the clinical usefulness of each score based on a continuum of potential thresholds for major bleeding events (*x*-axis) and the net benefit of using the score to stratify patients at risk (*y*-axis) comparing to assuming that no patient will suffer from major bleeding risk.

The calibration curve is shown in Figure 3. The ABC-bleeding risk score overestimated the major bleeding risk when comparing the predicted risk with the observed annulized event rate. DCA showed no difference in net benefit of ABC-bleeding risk score in identifying more major bleeding events without increasing the false positive rate compared with HAS-BLED score (Figure 4).

### Sensitivity analysis

In subgroups stratified by anticoagulation types, the incidence rate of major bleeding events in the warfarin subgroup was significantly higher than that in the NOAC subgroup (0.67%, 95%CI 0.44–0.98% *p* < 0.001 vs. 0.40%, 95%CI 0.25–0.60%, *P* < 0.001), the performance of the ABC-bleeding risk score was comparable with that of the modified HAS-BLED score [0.65 (95% CI: 0.53–0.76) vs. 0.64 (95% CI: 0.55–0.74), *P* = 0.93 for warfarin; 0.69 (95% CI: 0.58–0.80) vs. 0.62 (95% CI: 0.52–0.72), *P* = 0.28 for NOACs], as presented in Supplementary Table 3.

We further investigated the performance of ABC-bleeding risk and HAS-BLED scores by excluding the patients on antiplatelet therapy (*n* = 378, 13.1%), and the results were similar to our main findings (Supplementary Table 4). Discriminative performance of ABC-bleeding score was comparable with that of HAS-BLED score (0.69 vs. 0.64, *P* = 0.41); NRI also showed similar reclassification capacity of ABC-bleeding score compared with HAS-BLED score (15.5%, 95%CI −11.0–42.0%, *P* = 0.25); Although the IDI reached the significant threshold, it showed modest improvement when comparing ABC-bleeding score with HAS-BLED score (0.4%, 95%CI 0–0.8%, *P* = 0.02).

## Discussion

This observational study represented the first real-world Chinese-patient-specific study to validate the ABC-bleeding risk score in assessing major bleeding in patients with AF on OAC therapy. The GDF-15 level, an important variable included in the ABC-bleeding risk score, showed a significant association with major bleeding risk. Although we observed no significant difference in the C-index and reclassification capacity when comparing ABC-bleeding risk and the modified HAS-BLED scores, the further cross-table analysis suggested that these two scores were complementary and can cross-identified high-risk patients from the other’s low-risk group. Despite the overestimation of major bleeding risk, the ABC-bleeding score performed better in differentiating high-risk patients. Overall, we suggest that the ABC-bleeding risk score is an important tool in estimating major bleeding risk in Chinese patients with AF on OAC therapy, especially those at high risk. The two scores can complement one another to identify patients with a high risk of major bleeding for the further optimization of OAC therapy.

AF patients on OACs frequently develop bleeding events, the most severe of which can be fatal. Several factors, including age, uncontrolled hypertension, and renal failure, can enhance the patients’ risk of major bleeding. However, previous studies have demonstrated that the predictive performance of the risk assessing scores and tools based solely on clinical criteria is just moderate, especially for identifying individuals with a high risk of bleeding. Several biomarkers have been investigated as the subject for quantitatively assessing major bleeding risk. cTnT-hs, a biomarker reflecting cardiovascular endothelial integrity, was associated with an increased risk of major bleeding regardless of OACs patterns in the ARISTOTLE trial (19), and this association remained significant after adjusted cTnT-hs level by the components of the HAS-BELD score in the ENGAGE AF-TIMI 48 trial (14). However, the comparable predictive ability of cTnT-hs toward stroke makes it less specific in assessing bleeding risk. GDF-15, a marker of tissue hypoxia and oxidative stress, has received great attention in predicting major bleeding in patients on OAC (20). It has been observed in clinical trials that after controlling components in the HAS-BLED score and other biomarkers, including cTnT-hs and NT-proBNP, GDF-15 was highly related to the risk of major bleeding (10, 14). In our study, the significant association of GDF-15 with major bleeding risk was also recognized, regardless of adjusting the Cox-PH model using the components in the HAS-BLED score and cTnT-hs level. This observation further supports the previous studies in clinical trials and suggests the value of GDF-15 in indicating major bleeding risk.

Combining clinical factors and biomarkers related with major bleeding risk, a novel biomarker-based score, the ABC−bleeding risk score, was developed and utilized in estimating bleeding risk of patients with AF in the ARISTOTLE trial (12). Afterward, the outperformance of ABC-bleeding score compared to HAS-BELD score in patients receiving anticoagulation was externally validated in RE-LY and ENGAGE AF-TIMI 48 trials (14, 20). However, it should be noted that a previously reported real-world study concluded that the HAS-BLED score performed significantly better than the ABC-bleeding risk score, with a higher Harrell’s C-index (0.583 vs. 0.518) and positive NRI. This weaken in the predictive performance of the ABC-bleeding risk score in the established real-world study compare to in clinical trials may be attributed to several factors: Firstly, the differences in study design, demographic characteristics, and uncontrolled factors may lead to the variation in performance of the ABC-bleeding risk score; Secondly, the validation study in clinical trials implied that the risk discrimination performance of ABC-bleeding score was much better for AF patients on NOAC than on warfarin therapy, while at the baseline of the real-world study, patients only received warfarin therapy (21). Therefore, the performance of the ABC-bleeding score might be underestimated in the real-world study due to its unicity of OAC therapy; Thirdly, in the established real-world study, GDF-15 level was replaced by creatinine clearance estimated by the CKD-EPI equation, which might also weaken the performance of the ABC-bleeding risk score in predicting major bleeding events.

Unlikely the aforementioned studies in clinical trials and the real-world situation that both stating one score is significantly better than the other, a recent meta-analysis demonstrated that the HAS-BELD score is as least non-inferior to the ABC-bleeding score with a comparable c-index value (0.61 vs. 0.65, *P* > 0.05) (22), while further analyses investigating the discriminative ability of these two scores were limited. In addition, another network meta-analysis suggested that the HAS-BLED score has an optimal balance of sensitivity and specificity, while the ABC-bleeding score had comparatively higher sensitivity, defined as the ratio between the number of major bleeding events in high-risk stratification and the total number of bleeding events, suggesting that the ABC-bleeding score has its own strength in stratifying high-risk patients (23). Similar to these two meta-analyses, our real-world study suggested that the two scores did not perform significantly different in assessing major bleeding risk, and our further analysis revealed a better discrimination of the ABC-bleeding score to patients with a high risk of major bleeding. It should be recognized that several high risk patients could be further stratified by the HAS-BELD score from the groups with low-risk level identified by the ABC-bleeding score, and this might explain the non-significant difference between the two scores. Considering the validation of the two scores and the observed complementary effect in identifying high risk patients, a combination of the two scores might optimize the identification of patients with different risk levels.

Our study showed the potential clinical implications of ABC-bleeding score in real-world practice. When assessing the major bleeding risk of AF patients on OAC, physicians could use a combination of the ABC-bleeding risk score and the international guidelines recommended HAS-BELD score to recognize AF patients with a high risk of major bleeding. For the high-risk patients stratified by the two scores, international guidelines suggested that their high bleeding risk score should not lead to the withholding of OAC as the net benefit of OAC is even greater amongst such patients (3). However, the OAC treatment pattern of these patients will need careful management and active monitoring to prevent potential major bleeding events. GDF-15 and other biomarkers could also be used to monitor changes in risk indicators and could contribute to altering OAC therapy in AF treatment over time. In conclusion, we endorse that combining the ABC-bleeding risk and the modified HAS-BLED scores, as a comprehensive consideration of biomarkers and clinical information, could well recognize patients with a high risk of major bleeding and optimize the net benefit of OAC therapy in clinical practice.

## Limitations

The limitations of this study should be addressed. As a prospective observational study, baseline characteristics could be diverse among different bleeding risk levels in real-world situations, which might lead to bias in our results. Another limitation is that INR lability was not included in the calculation of the modified HAS-BLED score, as the TTR data for patients on warfarin were not available in our cohort. Although most patients were prescribed NOACs in our study, the absence of INR lability might cause a bias in assessing the predictive value of the HAS-BLED score. Moreover, as the cohort represents the Chinese population with AF on OAC, extrapolating the findings in this study to other ethnic groups requires further investigation. Finally, as the ABC-bleeding risk score overestimated the major bleeding risk due to the relatively lower event rate than previous studies, recalibration for better utilization requires further investigation.

## Conclusion

This observational study represented the first real-world validation of the ABC-bleeding risk score in China to assess major bleeding risk in patients with AF on OAC treatment. The predictive performance of the ABC-bleeding scores was not significantly different from the modified HAS-BLED score, and the ABC-bleeding risk score overestimated the major bleeding risk. but the ABC-bleeding score categories revealed a better capability in identifying high-risk patients. As the modified HAS-BLED score could stratify high-risk patients from the low-risk groups determined by the ABC-bleeding risk score, we endorse that combining the two risk scores would be a clinically practical strategy yielding a more comprehensive understanding of patients with different risk levels of major bleeding, especially for those at a high-risk level, and improving decision-making in OAC treatment.

## Data availability statement

The original contributions presented in this study are included in the article, further inquiries can be directed to the corresponding author.

## Ethics statement

The studies involving human participants were reviewed and approved by Human Research Ethics Committee of Beijing Anzhen Hospital. The patients/participants provided their written informed consent to participate in this study.

## Author contributions

Y-FW undertook study design and data analysis under the guidance of the MDs. Y-FW wrote this manuscript under the guidance of the corresponding author. All authors revised the article and approved the submitted version.
