# Supplementary material for: Performance of the ABC-bleeding risk score for assessing major bleeding risk in Chinese patients with atrial fibrillation on oral anticoagulation therapy: A real-world study
Source: Front Cardiovasc Med. 2022 Nov 3;9:1019986. doi: 10.3389/fcvm.2022.1019986 (PMC9669712; doi:10.3389/fcvm.2022.1019986)
Supplement: Supplementary file 1 [file Data_Sheet_1.docx]

# Supplementary Table 1. Cox proportional-hazards analysis of GDF-15 level in estimating major bleeding incidence.

|  | HR (95% CI) | *P* value | aHR* (95% CI) | *P* value |
| --- | --- | --- | --- | --- |
| log_2_(GDF-15 level) | 2.72 (1.68-4.41) | <0.001 | 2.16 (1.27-3.68) | 0.005 |

Abbreviations: CI, confidence interval; GDF-15, growth differentiation factor 15; HR, hazard ratio; aHR, adjusted hazard ratio

*GDF-15 was adjusted for components of HAS-BLED score and TnT-hs

**Supplementary Table 2.** Major bleeding incidence and Cox proportional-hazards regression analysis of low + medium and high-risk levels stratified by ABC-bleeding risk and modified HAS-BLED scores.

|  | **ABC-bleeding score risk level** | | **Modified HAS-BLED score risk level** | |
| --- | --- | --- | --- | --- |
|  | **Low + Medium** | **High** | **Low + Medium** | **High** |
| N (%) | 2,591  (89.59%) | 301  (10.41%) | 2,591  (89.59%) | 301  (10.41%) |
| No. | 33 | 15 | 38 | 10 |
| Incidence (% per year) | 0.39 | 1.49 | 0.45 | 0.97 |
| HR (95% CI) | - | 3.68  (1.96, 6.90) | - | 2.42  (1.20, 4.89) |
| *P* value | - | <0.001 | - | 0.01 |

Abbreviations: HR, hazard ratio; CI, confidence interval.

**Supplementary Figure 1.** Kaplan-Meier curve for major bleeding in patients at low- and medium-risk **(A)**, low- and high-risk **(B)**, and medium- and high-risk **(C)** stratified by the modified HAS-BLED scores.


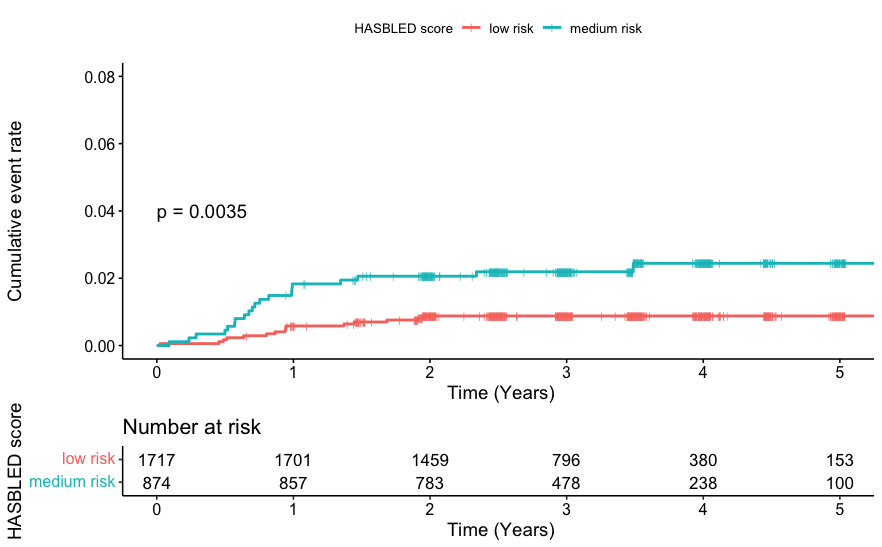


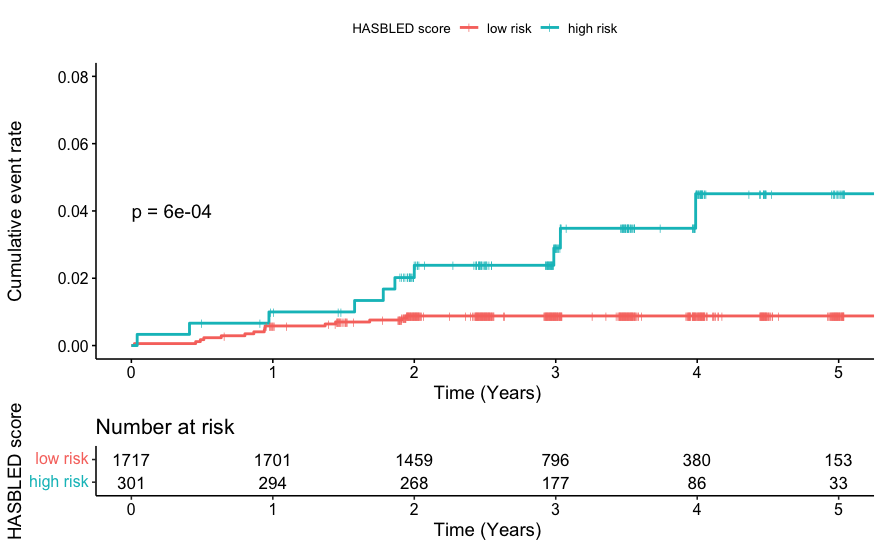


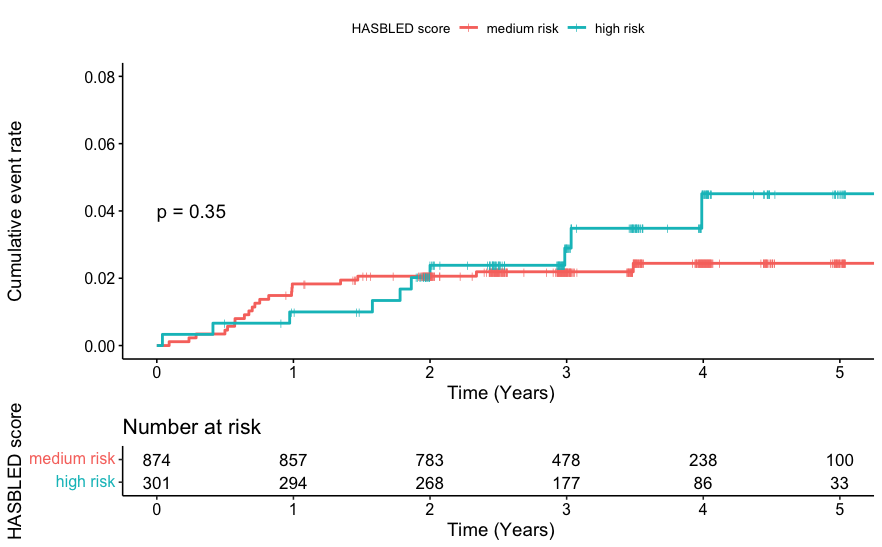


**Supplementary Figure 2.** Kaplan-Meier curve for major bleeding in patients at low + medium and high risk stratified by **(A)** the ABC-bleeding risk and **(B)** the modified HAS-BLED scores.


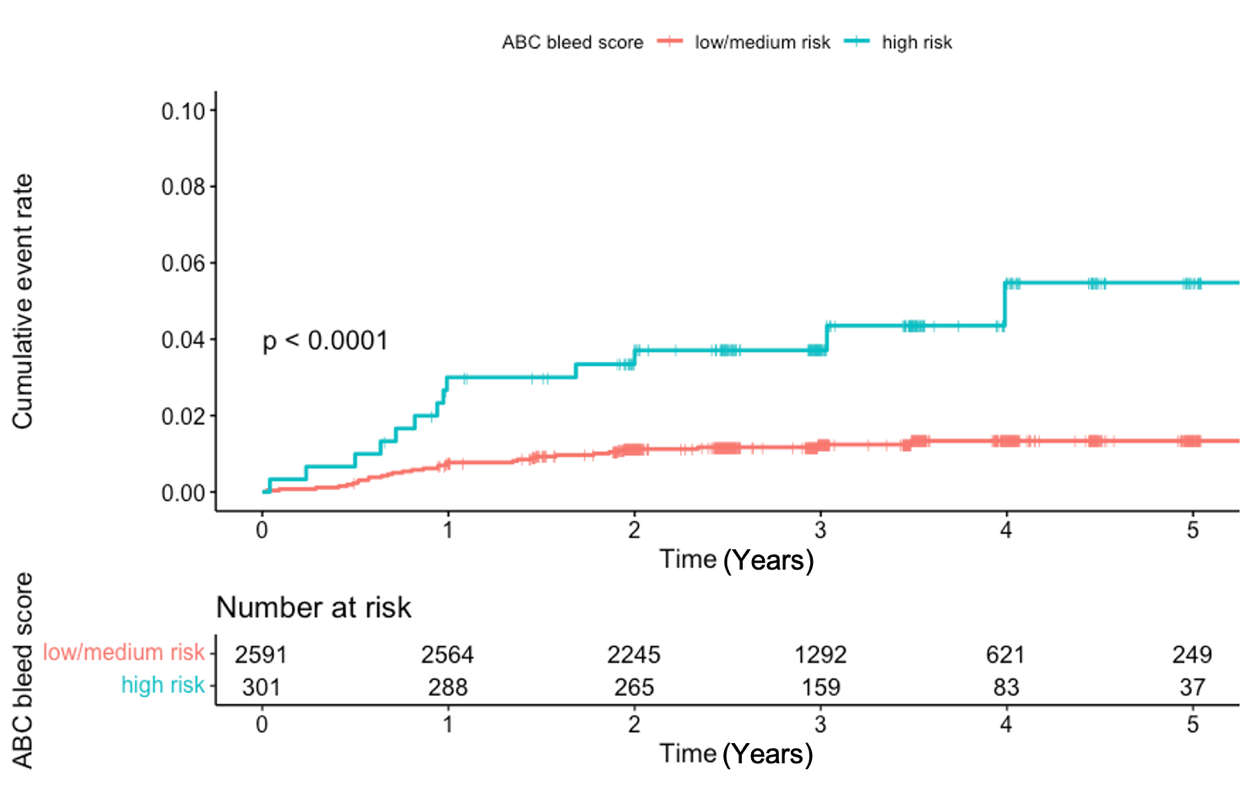


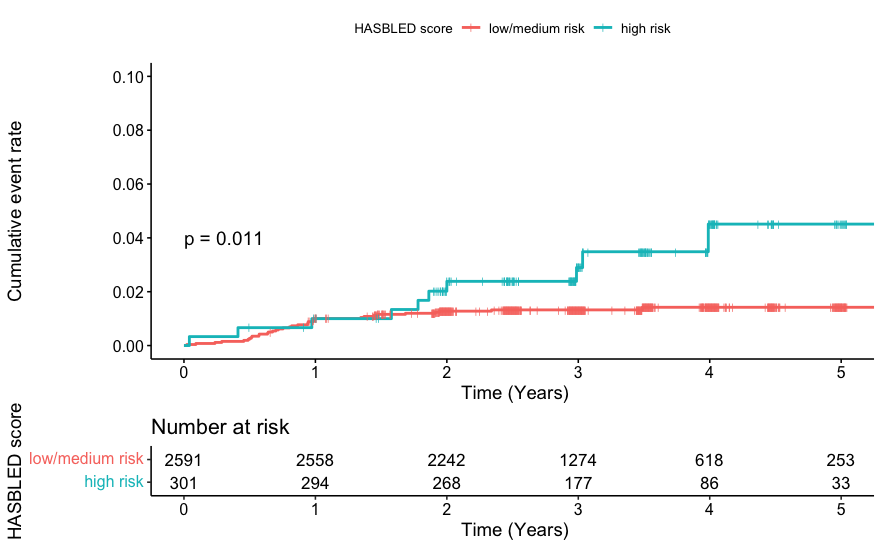


**Supplementary Table 3.** Harrell’s C-index of the ABC-bleeding risk and the modified HAS-BLED scores stratified by anticoagulants.

| **Anticoagulant** | **Predictive score** | **Harrell’s C-index** | **95% CI** | ***P* value*** |
| --- | --- | --- | --- | --- |
| Warfarin | ABC-bleeding risk | 0.65 | 0.53-0.76 | 0.93 |
|  | Modified HAS-BLED | 0.64 | 0.55-0.74 | - |
| NOAC | ABC-bleeding risk | 0.69 | 0.58-0.80 | 0.28 |
|  | Modified HAS-BLED | 0.62 | 0.52-0.72 | - |

Abbreviations: CI, confidence interval; NOAC, non-vitamin K antagonist oral anticoagulants, which includes three drugs: rivaroxaban, apixaban, and dabigatran; NRI, net reclassification improvement.

**P* value and NRI at Year 3 calculated versus the modified HAS-BLED score.

**Supplementary Table 4.** Discrimination and reclassification analysis of the ABC-bleeding risk and the modified HAS-BLED scores.

|  | **C-index** | **95% CI** | ***P* value*** | **NRI**  **at one year** | ***P***  **value** | **IDI**  **at one year** | ***P* value** |
| --- | --- | --- | --- | --- | --- | --- | --- |
| ABC-bleeding risk score | 0.69 | 0.60-0.77 | 0.41 | 15.5%  (-11.0%, 42.0%) | 0.41 | 0.4%  (0-0.8%) | 0.02 |
| Modified HAS-BLED score | 0.64 | 0.57-0.71 |  |  |  |  |  |

Abbreviations: CI, confidence interval; NRI, net reclassification improvement.

**P* value for comparison of ABC-bleeding score with the modified HAS-BLED score.
